# Breast Cancer Knowledge and Practice of Breast Self-Examination among Female University Students, Gaza

**DOI:** 10.1155/2021/6640324

**Published:** 2021-04-27

**Authors:** Samira S. Abo Al-Shiekh, Mohamed Awadelkarim Ibrahim, Yasser S. Alajerami

**Affiliations:** ^1^Ph.D. in Public Health, Head of Mammography Department, MOH, Gaza, State of Palestine; ^2^Department of Public Health, Faculty of Applied Medical Sciences, ALBaha University, KSA, Al Bahah, Saudi Arabia; ^3^Department of Medical Imaging, Al-Azhar University, Gaza, State of Palestine

## Abstract

Breast cancer is the highest public detected cancer among female population in the majority of countries worldwide. Breast self-examination (BSE) is a useful screening tool to empower women and raise awareness about their breast tissues and help detect any breast abnormalities when they occur. This study aimed to assess the level of female university students' knowledge and practice of BSE. A self-administered questionnaire was used to assess the knowledge about breast cancer and related items, and an observation checklist was used to test practicing BSE using a breast simulator. Eighty-six students participated in the study, 58.1% studying nursing and 41.9% studying clinical nutrition in the third (40.7%) or the fourth level (59.3%). Of them, 24.4% had previous family history of breast cancer. The majority of the students (80.2%) had previous information about breast cancer acquired from different sources, university studies (57%), the Internet (45%), and social media (41%). Findings showed good scores (≥70%) regarding signs and symptoms and risk factors of breast cancer; however, low knowledge scores (<70%) were detected regarding general knowledge about breast cancer disease, methods of early detection and management, and applying steps of practicing BSE. Roughly all the students (96.5%) have heard about BSE, and 69.8% knew the time to do BSE; however, only 31.4% practice it regularly. Three barriers to practice were dominant among students who do not have a breast problem (39.7%), do not know how to do it (37.9%), and being busy 31%. On the other hand, breast cancer early detection purpose and the presence of family history of breast cancer were considered facilitators to regular practice BSE. A statistically significant relationship existed between knowledge about the steps of applying the BSE and regular practicing. A training program should be implemented to increase the level of awareness about BC and practicing BSE.

## 1. Introduction

Breast Cancer (BC) is the most common detected cancer among women in the large mainstream (140 of 184) of countries worldwide [1]. More than 2 million new BC cases with a mortality rate 626.7 per 100,000 were recorded during the year 2018 [2].

It was reported that the Age-Standardized Incidence Rate of BC was 29.1/100,000 in Asia, 67.6/100,000 in the United States of America (USA), and 71.1/100,000 in Europe [1]. However, the mortality to incidence ratio is much higher with about 0.35 in Asia in comparison to 0.21 in the USA and 0.23 in Europe [1].

Breast cancer is the most common cancer among the female population in Gaza, making 34.8% of all cancer cases, and it is the second leading cause of cancer death after lung and bronchus cancer (13.9%) [3]. In accordance with the Cancer Registry in Gaza, 684 cases have been registered in the year 2016, making about 20.5% out of all cancer cases [4]. Besides, the most affected age groups were (45–54) years and (55–64) years, making about 23% and 24.6% of the BC cases, respectively [5]. Furthermor, the number of BC cases is expected to increase by 135% by 2040 [6].

Breast Cancer in women of young age remains a great challenge to patients, families, and healthcare providers. In spite of the fact that the diagnosis of BC is less common in women under 40, it can still have a larger effect than it is in older ones, as it is likely to appear at a later stage, having aggressive features [7, 8] and more aggressive treatment, chemotherapy and mastectomy, than patients in their forties [9].

In Palestine, breast screening methods such as CBE and mammography focus on women older than 40 or women over 35 with family history. Additionally, the sensitivity of mammography is decreasing with increased breast density [10]. Hence, females under this age should focus on other screening methods such as the Breast Self-Examination (BSE).

The World Health Organization does not recommend BSE as a screening tool; rather, it is useful to increase women awareness regarding their health [11]. The American Cancer Society recommends starting the BSE during high-school years on a monthly basis [12], as it is a serious phase of every adult woman's personal health regime.

Several studies conducted at several countries showed a fair and good knowledge about BSE among the university students; however, few of them perform BSE regularly. A study by Tewabe and Mekuria which included 222 female students at Bahir Dar University in North West Ethiopia showed 80.3% awareness level among the students, but the practicing was poor. Another study conducted at the University of Riyadh, Saudi Arabia, showed that, among the participants, 52.2% of the respondents had adequate overall knowledge toward BS and only 18% of all participants perform BSE [13]. Among Emirate students, 68.5% of participants were aware about BSE, but few participants actually performed BSE [14]. Furthermore, 33% of Turkish students perform BSE regularly [15]. In the Gaza Strip, there is a gap of information about the level of awareness among undergraduate students. Therefore, this research has been conducted to assess the level of university students' knowledge regarding Clinical Breast Examination (CBE), BC disease, BC signs and symptoms, BC risk factors, and mammography and to examine the steps and frequency of practicing BSE. Furthermore, the relationship between total knowledge and practicing BSE is also studied.

## 2. Methods and Materials

### 2.1. Study Design, Setting, and Period

A cross-sectional design was used to assess the level of knowledge and practicing BSE among the Faculty of Applied Medical Sciences (AMS) female students, Al-Azhar University, Gaza, Palestine. Data were collected from March 2019 to April 2019.

### 2.2. Participants

Students studying in nursing and clinical nutrition departments, academic levels III and IV, were invited to participate in the study. A total of 86 students participated in the study with a response rate 92.3%. A consent form was attached to the questionnaire to inform students about the study aim, assurance about the confidentiality of their information, and willingness to participate.

### 2.3. Study Instruments

Two tools were used to collect the data. The tools were arbitrated by experts, and their opinions were taken into consideration. Comments about ways for asking the questions and paraphrasing of some statements were required. After completion of the arbitration process, the instruments were translated into the Arabic version and revised by academic and clinical experts.

### 2.4. Self-Administered Questionnaire

After reviewing the literature, a self-administered questionnaire in the Arabic version was designed by the researcher to collect the necessary data. The questionnaire was divided into four parts; the first part consists of 13 questions and contains sociodemographic characteristics, age, address, marital status, number of household members, economic status, occupation, attendance of previous training courses, and family history of BC or breast-related diseases.

The second part included sixteen questions such as female university students' knowledge regarding BC definition, risk factors, signs and symptoms, methods of screening BC, methods of early detection and diagnosis of BC, stage of BC, and methods of treatment. In addition, there were questions about their knowledge and what they will do in case of developing BC.

The third part includes seventeen questions about students' knowledge regarding BSE, CBE, and mammography. The questions include students' knowledge about the definitions of terms, their importance, the best time to do these examinations, and the frequency to do the examinations.

The fourth part consists of six questions to ask the students about their knowledge regarding BSE, the best way to do BSE, frequency of practice, the time required to conduct the exam, and the main reasons that prevent students from practicing. This section is not examined during posttest 1 as practicing BSE is repeated one time every month.

The questionnaire was used to measure the female students' knowledge about BC, methods of screening, and frequency of practicing BSE.

### 2.5. Checklist and Simulator Used

An observational checklist was developed to observe the actual students' performance of BSE using a breast simulator (Dressing-Type Breast Tumor Check-up Breast Self-Examination Model). The simulation technique could enhance the clinical education of medical students and creates opportunities to practice new skills without the involvement of real patients [16].

The simulation technique was used previously in previous studies and in teaching processes. A hybrid simulation model, consisting of a silicone breast simulator jacket, was used in a previous related study [17]. Fifteen statements in the checklist including the required steps needed for BSE were used (position, site of examination, inspection, and palpation technique).

### 2.6. Statistical Analysis

The Statistical Package for Social Science (SPSS) program, version 24, was used to analyze the data. Knowledge score was calculated by recoding of the knowledge questions by giving one point to the correct answer and zero to the incorrect answer. Then, the points were summed, multiplied by 100 over the number of questions. Knowledge about risk factors was calculated by recoding of the 5-point Likert scale from one point for strongly disagreeing responses to five points for strongly agreeing responses and then multiplied by 100 over 70 (14 items *∗* 5 points). Inverse coding was performed for two risk factors (breast feeding and small breast) as they considered protective factors rather than risk. Knowledge about BC signs and symptoms was calculated by recoding of the 5-point Likert scale from one point for the strongly disagreeing responses to five points for strongly agreeing responses and then multiplied by 100 over 65 (13 items *∗* 5 points). We have four parts of students' knowledge; we calculated the total knowledge by summation of the score in each item and then multiplied by 100 over four.

Applying steps of practicing BSE, the score was calculated by recoding of the responses by one point for not done, two points for not done correctly, and three points for correctly done. Then, the points were summed for the fifteen items and then multiplied by 100 over 45 (15 items *∗* 3 points).

We categorize the knowledge and practice scores into two categories, good (if the score ≥70%) and low (if the score < 70%).

Statistical analysis performed included descriptive analysis and the chi-square test. The level of significance was considered at less than 0.05 and Confidence Interval (CI) at 95%.

### 2.7. Ethical Consideration

Permissions from the Palestinian Health Research Council (Helsinki Committee, PHRC/HC/510/19) and the dean of the Faculty of AMS through official letters were obtained. Anonymity of the students was maintained by obtaining their academic numbers rather than their names. All the participants had been informed about the study aim and its benefits, and their acceptance and agreement were obtained using a consent form.

## 3. Results

Table 1 shows the characteristics of the participated students. Eighty-six female students participated in the study who were studying at nursing (58.1%) or clinical nutrition (41.9%), third Level (40.7%) or fourth level (59.3%). The mean age of the students is 20.8 (min-max: 19–30). Roughly half of them live in Gaza City, and the rest of the students are living in the other four Gaza Governorates. The majority of the participants are not married. The majority of their parents have either a secondary education, 40.7% and 34.9% for mothers and fathers, respectively, or a university education, 50% and 40.7% for mothers and fathers, respectively. The mean of the number of their households is 7 members with monthly income 475 US dollars.

Regarding the family history of BC, 24.4% of the students had a family history and only five had previous breast problems, as shown in Table 2.

**Figure 1:**
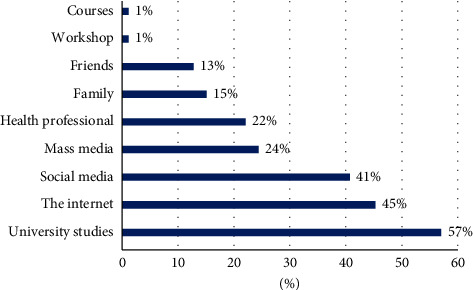
Sources of information about BC.

Figure 1 demonstrates the students' sources of information about BC. The majority of the students 69 (80.2%) claimed that they had previous information about BC which was acquired from different sources; the majority of these are university studies (57%), the Internet (45%), and social media (41%).

The result of knowledge scores among students is illustrated in Table 3. Regarding knowledge about BC disease, methods of detection, diagnosis, and management, the majority of the students (68.6%) have a low knowledge score with a mean 60.8 and SD 14.2.

More than half of the students (54.7%) have good knowledge score regarding risk factors of BC with a mean 72.4 and SD 6.3. In comparison, the majority of them (88.4%) have a good knowledge about signs and symptoms of BC with a mean 77.5 and SD 7.4.

With regards to participants' knowledge about mammography and CBE, only 23.3% of them have a good knowledge about this domain with a mean 64.9% and SD 12.5.

The total knowledge score about BC and BSE is low in general as only 44.2% of the participants have a good knowledge with a mean 68.9 and SD 6.5.

### 3.1. Practicing BSE

Table 4 presents information about the participants' practice of BSE. The majority of the students (96.5%) have heard about BSE. Also, the majority (69.8%) know the time to conduct BSE. However, only a third (31.4%) of the students claimed that they practice BSE regularly. Two reasons were pointed out by the students that encouraged them to adhere to practicing; the purpose of early detection of BC (85.2%) and the presence of family history (11.1%). On the other hand, the students revealed some barriers that hinder their practice of BSE. The most important barriers are not having a breast problem (39.7%), do not knowing how to do it (37.9%), and being busy (31%).

The study revealed that there is a statistically significant relationship between applying steps of BSE correctly and regular practicing in that 52.6% of students who have a good practicing score practice BSE regularly and 74.6% of those having a low practicing score do not practice BSE regularly (*χ*^2^ = 5.10, *p* value = 0.024). In addition, a positive trend was shown regarding the relationship between general knowledge about BC and regular practicing of BSE; however, the relationship is not statistically significant (*χ*^2^ = 3.11, *p* value = 0.078). On the other side, the findings of the current study do not show a relationship neither with the students' knowledge about signs and symptoms nor the BC signs (*p* value >0.05). The results are demonstrated in Table 5.

## 4. Discussion

Women in the Gaza Strip have a lot of challenges; one of them is fighting against BC, which is considered the most common cancer among them and the second leading cause of cancer deaths. Screening methods are essential in the early detection of BC and lead to decreased morbidity and mortality from cancer. It has been proposed that every woman should do the BSE starting from the age of 20 [18]. To our knowledge, there were no previous study conducted in Gaza to assess university students' level of knowledge and practice BSE; however, similar studies were conducted targeting women in different situations rather than students.

Students have knowledge about BC from different sources, the most common sources being university studies, the Internet, and social media. This finding is consistent with studies conducted in Egypt, Ethiopia, and Saudi Arabia universities that revealed the majority of medical students became aware of BC by their syllabuses [19–21].

The results of this study indicate that the students had relatively good knowledge about signs, symptoms, and risk factors of BC. The result is consistent with studies conducted in Egypt, Iraq, and Ethiopia [20–22]. The result of a previous study conducted in Gaza showed a good knowledge about BC risk factors among Gaza women; however, knowledge about signs and symptoms of BC was not satisfactory [23]. Another study recently conducted in Gaza and published showed poor recognition of cancer symptoms in general among adolescents and adult participants; however, their knowledge about cancer risk factors is good [24]. Additionally, the result is not in line with another study conducted in the Al-Quds University, Palestine, which included students from non-health-related disciplines showed poor identification of risk factors, signs, and symptoms of BC [25]. The difference in the findings of the current study and the previous studies is related to the different target populations as our participants' knowledge increased mainly because of their learning curriculum in the Faculty of AMS, and this supports another finding in this study that the most common sources of their information were derived from their university studies. Furthermore, the result enterprise calls to encourage mass media such as TV and radio to focus more about BC and related items to reach all women in different societies and localities.

The study revealed poor general knowledge about BC, early detection method and diagnosis, knowledge about CBE, and mammography. The result is inconsistent with three previous related studies. In their study, Jalambo and colleagues showed a good knowledge about mammography and CBE among women visiting PHCC. Another study is a master thesis study conducted in Gaza to assess the knowledge and practice BSE among nurses working at the primary healthcare centers, which showed a good knowledge about BC early detection methods, CBE, and mammography; however, their mammography practices were low [26]. In addition, [25] revealed a good knowledge about early detection methods of BC such as mammography and U/S.

The majority of participants heard about BSE and recognized that it is useful in early detection of BC disease. This finding is consistent with the literature [21, 23, 27]. Despite the students' awareness about BSE, the majority of them do not practice BSE regularly as only 31.4% do so, and the result is consistent with previous studies that showed poor practice of BSE which is between 17% and 30% among their participants [14, 21–23, 27]. About 70% of the participants know the time to conduct BSE, and this figure is much higher than that of university students in other countries, 16.5% among Egyptian students [20], 42.8% among Emirate students [14], and 48.5% among Palestinian students in the west bank [25]. This figure could be interpreted by their being study in paramedical sciences.

Students who regularly practice BSE showed that two important reasons encourage them to practice BSE, BC early detection purpose and the presence of a family history of BC. On the other hand, students who not regularly practice BSE face barriers that hinder their practice; the three most important recognized barriers are they do not have a breast problem, do not know how to do it, and being busy. The literature suggests barriers that discourage practicing BSE, most of which are not knowing how to do it, forgetting, and not interested by Rahman et al's study [14], unawareness of its importance and fear of results from Ahmed et al.'s study [20], lack of obligation and having a healthy breast in the study of Abdul-Lateef and Shabaan [22], not necessary and too busy [28], and no disease and lack of knowledge to do it [23, 27]. Of the facilitators reported in the literature is the routine examination for early detection of BC [28].

The study showed that there is no significant relationship between knowledge about BC and its related items with regularly practicing BSE. However, the literature suggested that adequate knowledge about BC was found to be significant predictors for BSE practice [29, 30]. The only factor which appears to affect their regular practicing in the current study is being knowledgeable about the steps of practicing. Therefore, it is crucial to teach female students to be aware about the importance and steps of practicing BSE.

### 4.1. Strengths and Limitations of this Study

To the best of our knowledge, this study is the first study conducted locally in Palestine and specifically in Gaza with the aim to assess the level of awareness and practice of undergraduate students regrading BSE. A simulator technique and an observation checklist are considered useful tools to examine the students' practice of BSE. This method is better than the self-reported scale which may lead to recall bias. However, using a sample from only one university is the major limitation of this study, which limits the generalizability of its results among all female students in Gaza.

## 5. Conclusions

Knowledge about BC disease and its related items is good in general. However, there is still a gap in information of issues related to early detection and management methods of BC. Moreover, the students have not been informed about the frequency and time of practicing BSE. Regular practicing of BC will be increased among the students if we teach them and inform them about the steps of practicing BSE. This indicates the importance of applying a training program to increase the level of awareness about BC and practicing BSE that comes within the local and international efforts fighting against this dangerous disease.

## Figures and Tables

**Table 1:** Baseline characteristics of study participants (*n* = 86).

Variable	*n* (%)	

*Specialty*		
Nutrition	36 (41.9)	
Nursing	50 (58.1)	

*Academic level*		
Third level	35 (40.7)	
Fourth level	51 (59.3)	

*Address*		
North Gaza	12 (14)	
Gaza	40 (46.5)	
Middle zone	16 (18.6)	
Khanyounis	13 (15.1)	
Rafah	5 (5.8)	

*Marital status*		
Single	75 (87.2)	
Married	11 (12.8)	

*What is the level of your mothers' education?*		
Less than secondary education	8 (9.3)	
Secondary education	35 (40.7)	
University education	43 (50)	

*What is the level of your fathers' education?*		
Less than secondary education	21 (24.4)	
Secondary education	30 (34.9)	
University education	35 (40.7)	

*Variable*	Mean ± SD	Min-max
Students' age	20.8 ± 1.3	19–30
Number of household members	7.1 ± 2.5	2–13
Monthly average household income (US dollars)	475 ± 368	0–2000

**Table 2:** Family and medical history.

Variable	*n* (%)
*Family history of BC*	
Yes	21 (24.4)
No	65 (75.6)

*Having a previous breast problem*	
Yes	5 (5.8)
No	81 (94.2)

**Table 3:** Level of knowledge and practice.

Domain	Domain description	*n* (%)	Min-mx	(%) Mean	SD
*Knowledge*					
General knowledge about BC	Low knowledge	59 (68.6)	29–64.7	60.8	14.2
	Good knowledge	27 (31.4)	70.6–88.2		
Knowledge about risk factors	Low knowledge	39 (45.3)	58.6–70	72.4	6.3
	Good knowledge	47 (54.7)	71.4–87.1		
Knowledge about signs and symptoms	Low knowledge	10 (11.6)	58.6–70	77.5	7.4
	Good knowledge	76 (88.4)	71.4–87.1		
Knowledge about mammography and CBE	Low knowledge	66 (76.7)	38.5–69.2	64.9	12.5
	Good knowledge	20 (23.3)	76.9–92.3		

*Total knowledge*	Low knowledge	48 (55.8)	51.3–69.9	68.9	6.5
	Good knowledge	38 (44.2)	70.0–85.9		

*Practicing BSE*					
Applying the steps of practicing BSE in a correct way	Low practice	67 (77.9)	33.3–68.9	52.2	23.2
	Good practice	19 (22.1)	71.1–100		

Level of knowledge about BC, risk factors, signs and symptoms, CBE, and mammography.

**Table 4:** Practicing BSE.

Variable	*N* (%)
*Have you heard about BSE?*	
Yes	83 (96.5)
No	3 (3.5)

*Do you practice BSE regularly?*	
Yes	27 (31.4)
No	59 (68.6)

*Time to practice BSE* (*a week after the start of menses*)	
Correct answer	60 (69.8)
Incorrect answer	26 (30.2)

*Reasons for practicing BSE* (*n* = 27)	
BC early detection	24 (85.2)
Presence of family history of BC	3 (11.1)

*Barriers hindering their practice of BSE*	
I do not have a breast problem	23 (39.7)
I do not know how to do that	22 (37.9)
I'm busy	18 (31)
I do not think I should	9 (15.5)
Too frequent to practice	8 (13.8)
I do not feel comfortable doing this	4 (6.9)
I'm afraid	4 (6.9)
I do not think it is necessary	1 (1.7)
I do not have a special	0 (0)

**Table 5:** Relationship between knowledge about BC and related items and regular practice of BSE.

Variable		Do you practice breast self-examination regularly? Preintervention		*χ* ^2^	*p* value
		No	Yes		
*General knowledge about BC*	Low knowledge	44 (74.6)	15 (25.4)	3.11	0.078
	Good knowledge	15 (55.6)	12 (44.4)		

*Knowledge about BC risk factors*	Low knowledge	30 (76.9)	9 (23.1)	2.29	0.16
	Good knowledge	29 (61.7)	18 (38.3)		

*Knowledge about BC signs and symptoms*	Low knowledge	7 (70.0)	3 (30.0)	0.01	0.615
	Good knowledge	52 (68.4)	24 (31.6)		

*Knowledge about mammography and CBE*	Low knowledge	44 (66.7)	22 (33.3)	0.49	0.482
	Good knowledge	15 (75.0)	5 (25.0)		

*Applying steps of BSE correctly*	Low practicing	50 (74.6)	17 (25.4)	5.10	0.024^*∗*^
	Good practicing	9 (47.4)	10 (52.6)		

## Data Availability

Data used to support the findings of this study are available from the corresponding author upon request.
